# Clarithromycin is an effective immunomodulator when administered late in experimental pyelonephritis by multidrug-resistant *Pseudomonas aeruginosa*

**DOI:** 10.1186/1471-2334-6-31

**Published:** 2006-02-21

**Authors:** Evangelos J Giamarellos-Bourboulis, Anastasia Antonopoulou, Maria Raftogiannis, Pantelis Koutoukas, Thomas Tsaganos, Vassiliki Tziortzioti, Charalambos Panagou, Theodoros Adamis, Helen Giamarellou

**Affiliations:** 1 4th Department of Internal Medicine, University of Athens, Medical School, Greece

## Abstract

**Background:**

To apply clarithromycin as an immunomodulatory treatment in experimental urosepsis by multidrug-resistant *Pseudomonas aeruginosa*.

**Methods:**

Acute pyelonephritis was induced in 40 rabbits after inoculation of the test isolate in the renal pelvis. Therapy was administered upon signs of sepsis in four groups: A, controls; B, intravenous clarithromycin; C, amikacin; and D, both agents. Survival and vital signs were recorded; blood was sampled for culture and estimation of pro-inflammatory mediators; monocytes were isolated for determination of apoptotic rate and ex vivo TNFα secretion. Quantitative cultures and biopsies of organs were performed after death.

**Results:**

Increased rectal temperature and oxygen saturation were found in groups B and D compared to A and C. Mean survival of groups A, B, C and D was 2.65, 7.15, 4.25 and 8.70 days respectively. No differences were noted between groups concerning bacterial load in blood and tissues and serum endotoxins. Serum MDA and total caspase-3 activity of monocytes of group D decreased following treatment compared to other groups. Negative correlation was detected between cytoplasmic caspase-3 and ex vivo secretion of TNFα of blood monocytes of group A; similar correlation was not found for any other group. Pathology scores of liver and lung of group B were lower than group A.

**Conclusion:**

Clarithromycin administered late in experimental urosepsis by multidrug-resistant *P. aeruginosa *prolonged survival and ameliorated clinical findings. Its effect is probably attributed to immunomodulatory intervention on blood monocytes.

## Background

Thorough search for the evolution of methods of immunointervention in sepsis is based on animal models of pre-treatment. In these models therapy is administered before induction of sepsis; as a consequence their findings signify the importance of the administered compound to inhibit triggering of the inflammatory cascade [1]. On the contrary, in clinical practice, patients are admitted to hospitals due to sepsis resulting from a full-blown inflammatory cascade; in these patients immunomodulators have failed probably because they were not potent enough to inhibit an inflammatory cascade that had already been initiated [2]. Intravenous clarithromycin has already been applied as pre-treatment in experimental pyelonephrits by multidrug-resistant *Pseudomonas aeruginosa *leading to prolonged survival and attenuation of the systemic inflammatory response [3]. The effect of clarithromycin was attributed to the elaboration of serum concentrations close to 10 μg/ml, where nuclear factor-κB (NF-κB), the transcription factor of pro-inflammatory cytokines, is inhibited [4].

The present study attempted to simulate clinical practice; therefore clarithromycin was administered upon presentation of clinical signs of urosepsis in an experimental animal model. Aims of the study were: a) the effect of clarithromycin on overall survival; b) its probable synergy with amikacin, and; c) its effect on blood monocytes.

## Methods

### Bacterial isolate

One blood isolate of *P. aeruginosa *derived from a female patient with acute pyelonephritis and severe sepsis was applied. Minimal inhibitory concentrations (MICs) of ticarcillin/clavulanate, piperacillin, ceftazidime, imipenem, meropenem, ciprofloxacin, clarithromycin and amikacin determined by a microdilution technique were equal to >256/2, >512, 16, 64, 32, >512, 128 and 256 mg/L respectively; the isolate was considered multidrug-resistant [5].

The isolate was stored as multiple aliquots in skim milk (Oxoid Ltd, London, UK) at -70°C. Before each experiment, one aliquot was thawed and cultured onto McConkey agar plates (Becton Dickinson, Cockeysville Md). Single colonies were suspended in Mueller-Hinton broth (Oxoid) and incubated for 12 hours at 37°C in a shaking water bath. The resulting inoculum was washed three times with NaCl 0.9% to remove any free endotoxins.

### Animals

A total of 40 white New Zealand male rabbits of a mean (± SD) weight of 3.19 ± 0.30 kg were applied. The study received permit from the Veterinary Directorate of the Perfecture of Athens according to the Greek legislation in conformance to the 160/1991 Council Directive of the EU. Animals were housed in single metal cages and had access to tap water and standard balanced rabbit chow *ad libitum*. Room temperature ranged between 18 and 22°C, relative humidity between 55 and 65% and the light/dark cycle was 6 am/6 pm.

### Study design

Acute pyelonephritis was induced by a modification of the protocol, described by others [3,6]. Animals were initially sedated by the intramuscular injection of 25 mg/kg of ketamine and 5 mg/kg of xylazine. Anesthesia was maintained by the intramuscular administration of 15 mg/kg of xylazine at 30-minute time intervals. Through an upper midline abdominal incision, the peritoneal cavity was entered and the intestines were displaced to the left. The right ureter was recognized and ligated with a 3.0 suture just below the pelvis. A total of 1 × 10^7 ^cfu of the pathogen, at a volume of 0.1 ml, were injected by a 26-Gauge needle into the renal pelvis, proximal to the suture. The peritoneal cavity and the abdominal wall were closed in layers.

Vital signs of the animals were recorded at 30-minute time intervals; oxygen saturation by an electrode attached to the left ear (Electronic for Medicine); and body temperature by a thermometer placed in the rectum (Omron, Berlin, Germany). One ml of blood was sampled by puncture of the vein of their left ear and collected into EDTA-coated tubes (Vacutainer, Becton Dickinson) before and 1.5 hours after bacterial challenge. White blood cell counts were estimated by a standard analyzer. Hypothermia, decrease of oxygen saturation and leukopenia were noted within 1.5 hours after bacterial challenge (see below). These signs were indicative of the advent of sepsis [7], so as to allow for that time interval to be considered as adequate for the administration of treatment.

Animals were then divided into four study groups as follows:

- Group A (n: 10); controls administered 30 ml of normal saline intravenously.

- Group B (n: 10); animals administered two doses of clarithromycin. It was provided as a pyrogen-free amorphous powder (Abbott, Chicago, USA). After reconstitution with 10 ml of 5% glucose, appropriate amounts were added into NaCl 0.9% at a final volume of 30 ml that was infused by a pump within 30 minutes. The first dose, equal to 80 mg/kg, was administered 1.5 hours after bacterial challenge; the second dose, equal to 30 mg/kg, 3.5 hours after bacterial challenge. Each dose was infused within 30 minutes, in analogy to former studies [3].

- Group C (n: 10); animals administered intravenous bolus 8 mg/kg of amikacin, two hours after bacterial challenge, as proposed elsewhere [8].

- Group D (n: 10); animals administered both clarithromycin and amikacin; the first dose of clarithromycin was followed by amikacin.

A volume of 3 ml of blood was sampled from the vein of the left ear of each animal before the operation, and at 1.5, 3.5, 24 and 48 hours and collected into pyrogen-free tubes (Vacutainer, Becton Dickinson). After centrifugation, serum was kept refrigerated at -70°C until assayed for endotoxins (LPS), tumor necrosis factor-alpha (TNFα), malondialdehyde (MDA) and drug levels. At 3.5, 24 and 48 hours one more ml of blood was collected into heparin-coated syringes for the isolation of monocytes.

Survival of animals was recorded each 12 hours for a total period of follow-up of 21 days. After death autopsy was performed; animals remaining alive after 21 days of follow-up were sacrificed by the intravenous administration of sodium thiopental. Under sterile conditions, segments from the right kidney, liver, spleen and lower lobe of the right lung were taken and placed into separate sterile plastic containers for quantitative cultures and biopsy.

### Assays for LPS, TNFα and MDA

For the estimation of LPS, serum samples were diluted 1:10 in sterile and pyrogen-free water (BioWhitaker, Walkersville, Maryland, USA) and incubated for five minutes at 70°C. The concentration of LPS was then measured by the QCL-1000 Limulus Amoebocyte Lysate assay (BioWhitaker, lower limit of detection 1 EU/ml) using a standard curve created by known concentrations of LPS by *Escherichia coli *serotype O111:B4. All determinations were performed in duplicate and the mean of two observations was applied.

TNFα was measured by a bioassay on L929 fibrosarcoma cell line, as already described [3,9]. Briefly, confluent cells were thoroughly washed with Hank' s solution and harvested with 0.25% thrypsin/0.02% EDTA (Biochrom AG, Berlin, Germany). Cells were centrifuged, re-suspended in RMPI 1640 supplemented with 10% Fetal Bovine Serum and 2 mM of glutamine (Biochrom AG) and distributed into a 96-well cell culture plate at a density of 1 × 10^5 ^cells/well. The final volume of fluid into each well was 0.05 ml. After incubation for 2–3 hours at 37°C at 5% CO_2_, 0.06 ml of serum or of standard dilutions of known concentrations of human TNFα (Sigma, range 5.75–375.00 pg/ml) were added into each well followed by 0.05 ml of a 0.3 mg/ml dilution of cycloheximide (Sigma). Incubation continued over-night; then the supernatant of each well was discarded by aspiration and 0.1 ml of a 0.5 mg/ml methylene blue solution in methanol 99% was added. After ten minutes, the dye was removed and wells were thoroughly washed three times with 0.9% sodium chloride. Wells were left to dry and remnants of the dye in each well became soluble by the addition of 0.1 ml of 50% glacial acetic acid (Merck, Darmstadt, Germany). Optical density in each well was read at 495 nm (Hitachi Spectophotometer, Tokyo, Japan) against blank wells and control wells without added serum. Concentrations of TNFα were estimated by the reduction of the optical density of control wells by unknown samples applying a standard curve generated by standard concentrations. All determinations were performed in quadruplicate. The inter-day variation of the assay was 13.75%.

Lipid peroxidation was estimated by the concentration of MDA, as already described [10]. Briefly, a 0.1 ml aliquot of each sample was mixed to 0.9 ml of trichloroacetic acid 20% (Merck) and centrifuged at 12,000 g and 4°C for 10 minutes. The supernatant was removed and incubated with 2 ml of thiobarbituric acid 0.2% (Merck) for 60 minutes at 90°C. After centrifugation, a volume of 10 μl of the supernatant was injected into a high-performance liquid chromatography system (HPLC, Agilent 1100 Series, Waldbronn, Germany) with the following characteristics of elution: Zorbax Eclipse XDB-C18 (4.6 × 150 mm, 5 μm) column under 37°C; mobile phase consisting by a 50 mM K_3_PO_4 _(pH: 6.8) buffer and methanol 99% at a 60/40 ratio with a flow rate of 1 ml/min; fluorometric detection with signals of excitation at 515 nm and emission at 535 nm. The retention time of MDA was 3.5 minutes and it was estimated as μmol/l by a standard curve created with 1, 1, 3, 3-tetramethoxy-propane (Merck). All determinations were performed in duplicate. The total oxidant status of each animal was assessed by the area under the MDA versus time curve; it was calculated by the linear trapezoidal rule and expressed in μmol.h/l.

### Blood and tissue cultures

Volumes of 0.5 ml of blood were added into glass tubes with two ml of thioglycolate medium (Becton Dickinson). A 0.1 ml aliquot was then diluted 1:10 into sterile sodium chloride four consecutive times. Another aliquot of 0.1 ml of each dilution was plated onto McConkey agar and incubated at 35°C for a total period of three days. Plates were incubated at 35°C and the number of viable colonies were counted into each dilution and multiplied by the appropriate dilution factor. The lower detection limit was 50 cfu/ml. Bacterial cells in blood were expressed by their log_10 _value.

Tissue segments were weighted and homogenized; a 0.1 ml aliquot was diluted, plated and incubated as described for blood samples. Identification of colonies was performed by the API20E system (bioMérieux, Paris, France). The number of viable cells was expressed as its log_10 _value in cfu/g. The lower detection limit was 10 cfu/g

### Assay for blood monocytes

For the isolation of blood monocytes, heparinized venous blood was layered over Ficoll Hypaque (Biochrom, Berlin, Germany) and centrifuged. The separated mononuclear cells were washed three times with PBS (pH 7.2) and re-suspended in RPMI 1640 supplemented with 10% FBS and 2 mM of glutamine in the presence of 100 U/ml of penicillin G and 0.1 mg/ml of streptomycin (Sigma). After incubation for 1 hour at 37°C in 5% CO_2_, non-adherent cells were removed while adherent monocytes were washed three times with Hank' s solution. Cells were then harvested by 0.25% trypsin/0.02% EDTA and counted. Half of them were treated with an ice-cold cell lysis buffer (50 mM HEPES, 0.1% CHAPS, 5 mM DTT, 0.1 mM EDTA, pH 7.4). After centrifugation for ten minutes at 10,000 g under 4°C, activity of caspase-3 was estimated in the cytosolic extract by an enzymatic chromogenic assay (BIOMOL Research Laboratories, Plymouth, PA). It was based on the rate of hydrolysis at 37°C of a substrate releasing p-nitroaniline over-time, as assessed by sequential photometry at 410 nm. The assay was also performed in the presence of a caspase-3 inhibitor. The activity of caspase-3 in cell extracts was expressed as pmol/min.10^4 ^cells. The total caspase-3 activity of monocytes of each animal was assessed by the area under the caspase-3 versus time curve; it was calculated by the linear trapezoidal rule and expressed in pmol/10^4 ^cells.

The remaining half of monocytes were added into 12-well plates at a volume of 2.4 ml per well with RPMI 1640 supplemented with 10% FBS and 2 mM of glutamine. They were incubated for 18 hours at 37°C under 5% CO_2_. TNFα of supernatants was estimated, as described above, and expressed in pg/10^4 ^cells.

### Pharmacokinetic analysis

Concentrations of clarithromycin in serum samples were estimated by a modification of the method described elsewhere [11]. Briefly, 0.1 ml of serum were mixed with 0.1 ml of a saturated dilution of NaCO_3 _and 1 ml of isopropanol (Merck) and stirred for 15 minutes at 400 rpm. The supernatant was centrifuged for 5 minutes at 2,700 g and the organic layer was evaporated. The remaining was dissolved in 0.1 ml of acetonitrile and incubated for 40 minutes at 40°C with 0.1 ml of a K_3_PO_4 _buffer (100 mM, pH 6.8) and 0.1 ml of a 2.5 mg/ml dilution of FMOC (9-fluorenylmethyloxycarbonyl chloride, Sigma) in acetonitrile. A volume of 50 μl was then injected into an HPLC system with the following characteristics of elution: Nucleosil 100-5 C18 (4.0 × 250 mm, 5 μm) column under 37°C; mobile phase consisting by a 50 mM K_3_PO_4 _(pH: 4.0) buffer and acetonitrile at a 60/40 ratio with a flow rate of 1 ml/min; ultraviolet detection at 210 nm. One standard curve of known concentrations was applied with erythromycin as an internal standard. The lower detection limit was 0.19 mg/l.

Concentrations of amikacin were estimated by a microbiological assay with *Escherichia coli *ICB4004 as a reference strain. Concentrations were estimated after designing a standard curve with known concentrations into semilogarithmic climax. The lower detection limit was 0.7 mg/l.

### Tissue pathology

Tissue segments were fixed with formalin, sliced appropriately, embedded in paraffin and stained with hematoxylin and eosin. A semi-quantitative scoring system was used where segments of liver, kidney and lung were separately scored for acute and chronic inflammation. Both acute inflammation, characterized by infiltration by neutrophils, and chronic inflammation characterized by the presence of mononuclear cells were scored as 0 (absent), 1 (sparse), 2 (moderate) and 3 (high). Segments of spleen were separately scored for the enlargement of B- and T-lymphocyte areas (0: absent, 1: slight, 2: moderate, and 3: pronounced) and for the presence of germinal centers (0: absent, 1: presence). One single value was applied for each organ segment after addition of separate scores (lowest value: 0; highest value: 6).

### Statistical analysis

Results were expressed by their mean (± SE). Comparisons between groups were performed by Mann Whitney; values were adjusted according to Bonferroni to avoid any random correlation. Comparisons concerning tissue bacterial load and pathology scores involved only those animals that died. Survival was estimated by Kaplan-Meier analysis; groups were compared by log-rank test.

Correlation between monocyte caspase-3 activity and ex vivo production of TNFα by monocytes, as well as between organ bacterial load and pathology scores were performed according to Spearman.

Any value of p below 0.05 was considered as significant.

## Results

**Figure 1:**
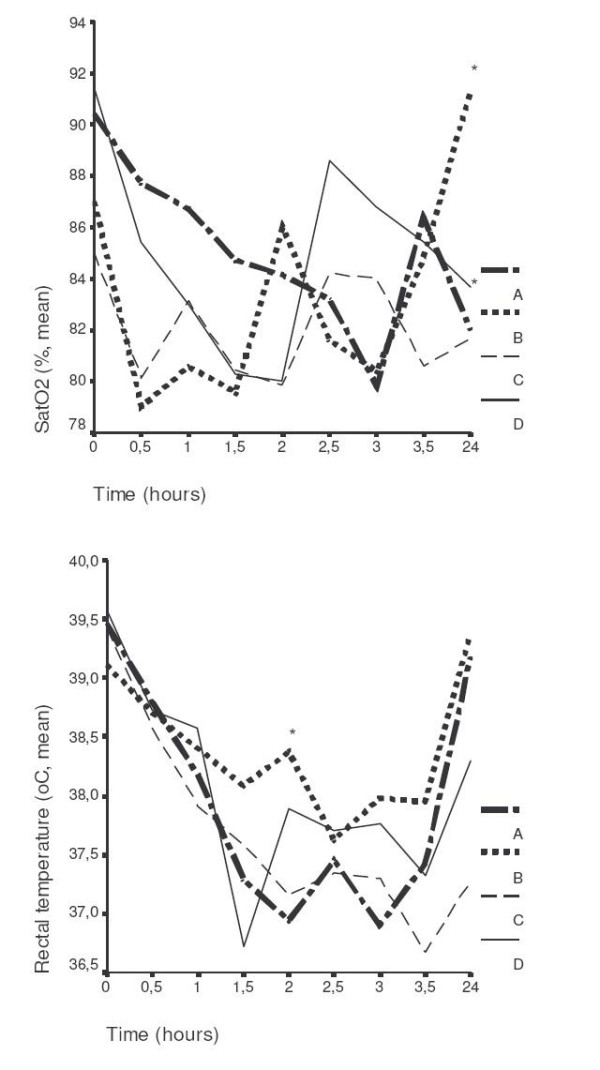
Comparative values of oxygen saturation and rectal temperature of animals with acute pyelonephritis by multidrug-resistant *Pseudomonas aeruginosa*. A: controls, B: clarithromycin, C: amikacin, D: both agents. Therapy was started 1.5 hours after bacterial challenge; asterisks denote statistical differences compared to group A.

Comparative curves of rectal temperature and oxygen saturation over-time of groups of treatment are shown in Figure 1. Mean (± SE) of white blood cells of all animals before operation were 9,230.0 ± 565.0/μl. At 1.5 hours after bacterial challenge they were 6,157.5 ± 684.6/μl, 5,740.0 ± 618.1/μl, 5,500.0 ± 817.3/μl and 6,142.8 ± 468.5/μl for groups A, B, C and D respectively.

**Figure 2:**
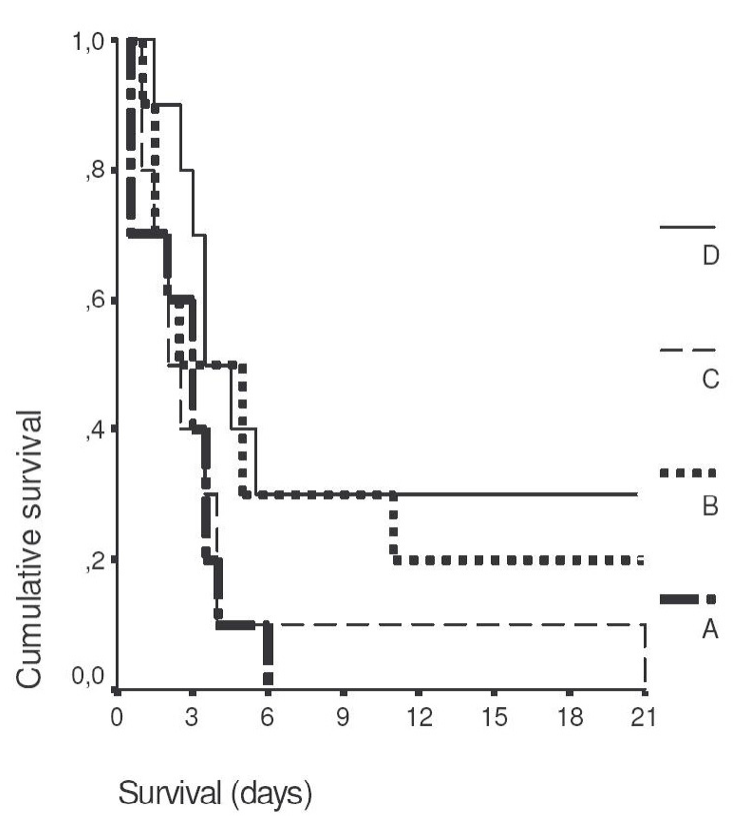
Comparative survival of animals with acute pyelonephritis by multidrug-resistant *Pseudomonas aeruginosa *and administration of therapy upon presentation of signs of sepsis. A: controls, B: clarithromycin, C: amikacin, D: both agents. p of comparisons between groups: A vs D 0.049; C vs D 0.045; A vs C NS (non-significant); and A vs B NS

Over the 21-day follow-up, death occurred in all animals of group A (mortality 100%); in eight animals of group B (mortality 80%); in all animals of group C (mortality 100%); and in seven animals of group D (mortality 70%). Mean (± SE) survival of animals of group A was 2.65 ± 0.57 days, of group B 7.15 ± 2.36 days, of group C 4.25 ± 1.89 days and of group D 8.70 ± 2.57 days. Survival curves of all groups of treatment are shown in Figure 2. Statistically significant differences were found between groups A and D (p: 0.049), between groups C and D (p: 0.045) but not between groups A and C neither between groups A and B.

Concentrations of bacteria in blood, LPS, MDA and TNFα detected in serum over follow-up of groups A, B, C and D are given in Table 1. Total oxidant status of groups A, B, C and D as assessed by the area under the MDA versus time curve were 161.89 ± 28.88, 119.51 ± 18.41, 135.87 ± 44.22 and 101.21 ± 37.64 μmol.h/l respectively. Significant differences were found only between groups A and D (p: 0.021).

**Table 1:** Comparative concentrations of bacteria on blood, endotoxins (LPS), malondialdehyde (MDA) and tumor necrosis factor-alpha (TNFα) of groups of treatment. Therapy was administered 1.5 hours after bacterial challenge.

**Time (hours) after bacterial challenge**	**A (controls)**	**B (clarithromycin)**	**C (amikacin)**	**D (clarithromycin + amikacin)**
	
	**Bacteria in blood (Mean ± SE, log_10 _cfu/ml)**			
1.5	1.72 ± 0.31	2.53 ± 0.61	1.92 ± 0.23	2.00 ± 0.75
3.5	1.95 ± 0.26	2.05 ± 0.36	2.22 ± 0.36	1.20 ± 0.20
24	3.35 ± 1.23	4.91 ± 0.99	6.24 ± 0.76	3.72 ± 0.89
48	5.93 ± 1.06	5.94 ± 1.06	5.23 ± 1.77	5.13 ± 0.93
	**LPS (Mean ± SE, EU/ml)**			
1.5	10.79 ± 7.28	4.53 ± 1.94	10.15 ± 6.87	5.88 ± 2.68
3.5	1.17 ± 0.54	6.25 ± 2.94^a^	10.02 ± 6.72	3.92 ± 0.73
24	7.35 ± 2.02	11.09 ± 4.87	9.88 ± 3.72	36.22 ± 5.52
48	21.86 ± 16.42	3.00 ± 1.24	4.81 ± 2.79	8.11 ± 6.97

	**MDA (Mean ± SE, μmol/l)**			
0	2.56 ± 0.26	3.02 ± 0.88	2.18 ± 0.78	1.50 ± 0.39
1.5	2.52 ± 1.05	4.57 ± 1.33	3.48 ± 1.48	1.87 ± 0.52
3.5	3.19 ± 1.06	4.54 ± 1.26	2.09 ± 1.22	1.29 ± 0.26
24	3.74 ± 1.00	1.71 ± 0.73^b^	2.72 ± 2.24	1.89 ± 0.53
48	1.75 ± 0.53	3.13 ± 1.44	3.86 ± 2.79	4.77 ± 3.68

	**TNFα (Mean ± SE, pg/ml)**			
0	18.91 ± 4.95	21.98 ± 8.04	21.99 ± 4.71	22.50 ± 9.09
1.5	34.53 ± 15.55	11.57 ± 0.08	40.96 ± 23.29	113.67 ± 55.52
3.5	43.72 ± 27.02	17.33 ± 5.82	643.85 ± 609.53	31.77 ± 20.51
24	20.81 ± 11.71	248.82 ± 226.51	29.46 ± 17.96	60.49 ± 50.65
48	977,62 ± 97,63	32.81 ± 13.23	18.11 ± 6.61	47.53 ± 26.46

*P *of comparisons to group A: ^a^0.042, ^b^0.032

The cytoplasmic activity of caspase-3 of blood monocytes and the ex vivo release of TNFα by blood monocytes of each group is given in Table 2. The total caspase-3 activities of monocytes of groups A, B, C and D as assessed by the area under the caspase-3 versus time curve were 15,350.5 ± 9,234.5, 13,388.3 ± 11,252.7, 1,015.9 ± 536.8 and 473.7 ± 175.8 pmol/10^4 ^cells respectively. Significant differences were found only between groups A and D (p: 0.048). A negative correlation was detected between caspase-3 activity and ex vivo release of TNFα of monocytes of group A (r: -0.466, p: 0.044). A lack of significance was noted regarding the respective correlation in groups B (r: +0.344, pNS), C (r: -0.179, pNS) and D (r: -0.080, pNS).

**Table 2:** Comparative rate of apoptosis and biosynthetic activity of blood monocytes of four animal groups with acute pyelonephritis by multidrug-resistant *Pseudomonas aeruginosa*. Rate of apoptosis was expressed by the activity of cytoplasmic caspase-3; biosynthetic activity by the ex vivo release of tumor necrosis factor-alpha (TNFα). Therapy was administered 1.5 hours after bacterial challenge.

**Time (hours) after bacterial challenge**	**A (controls)**	**B (clarithromycin)**	**C (amikacin)**	**D (clarithromycin + amikacin)**
	
	**Cytoplasmic activity of caspase-3 (Mean ± SE, pmol/min.10^4 ^cells)**			
3.5	1810.2 ± 1779.6	3210.3 ± 1173.5	277.5 ± 273.0	1155.7 ± 647.8
24	2187.0 ± 1205.9	1096.6 ± 431.9	663.7 ± 662.2	647.9 ± 453.1
48	1129.5 ± 863.3	908.1 ± 908.1	6575.5 ± 657.8	112.3 ± 80.6
	***Ex vivo *release of TNFα (Mean ± SE, pg/10^4 ^cells)**			
3.5	96.6 ± 61.9	394.6 ± 315.5	132.2 ± 44.3	342.7 ± 259.9
24	60.5 ± 39.9	34.5 ± 15.8	143.3 ± 126.5	10.6 ± 3.9^a^
48	63.1 ± 25.1	15.9 ± 10.6^b^	79.4 ± 68.4	71.7 ± 19.1

*P *of comparisons to group A: ^a^0.016, ^b^0.049

**Figure 3:**
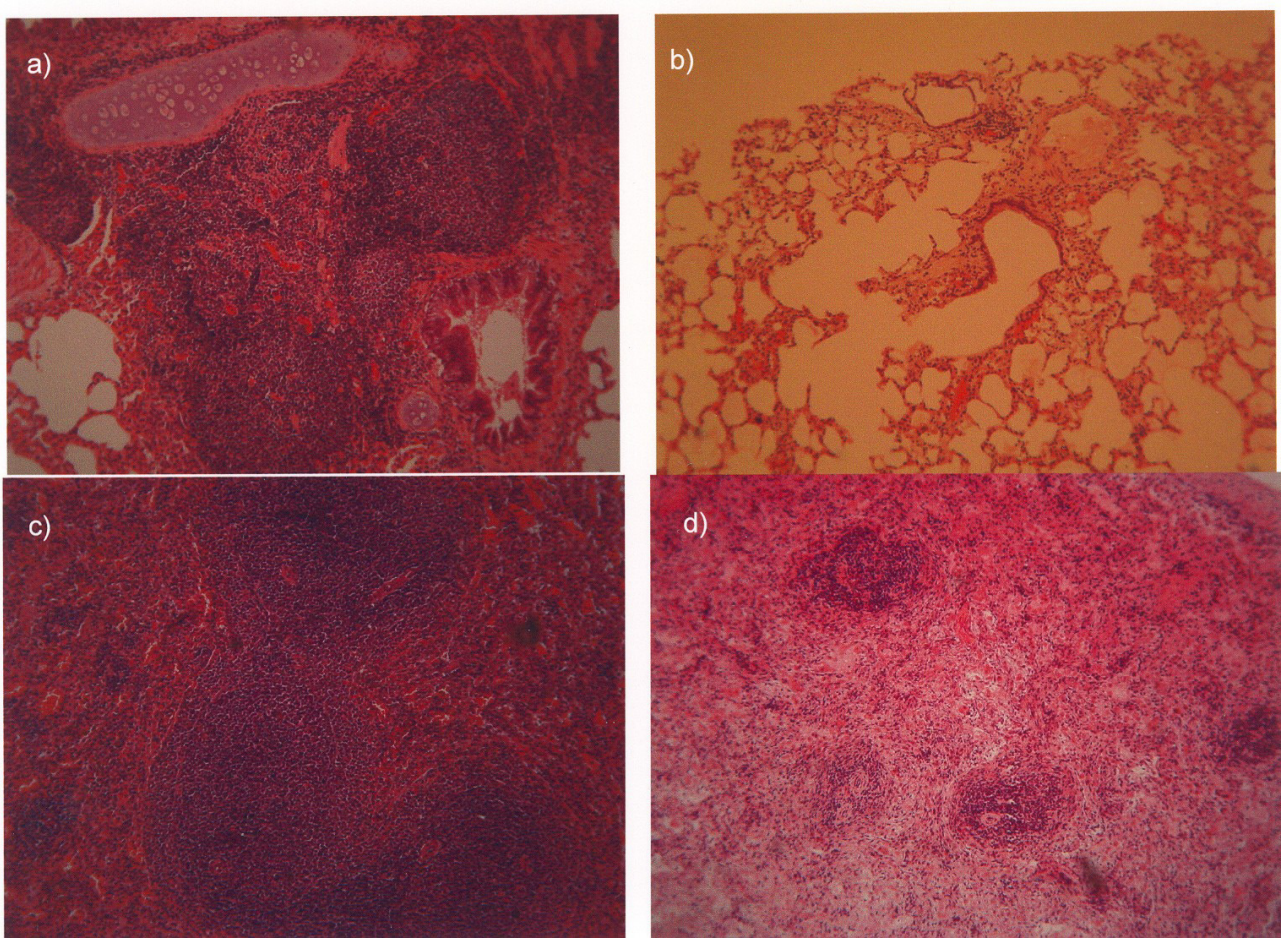
(legend to color Figure). (a) Section of lung with intense peribronchial inflammation of one animal of group A-controls that died 1.5 days after bacterial challenge and; (b) showing very mild interstitial inflammation of one animal of group B-administered clarithromycin that died 2 days after bacterial challenge. (c) Section of spleen with intense activation of B- and T-lymphocyte dependent areas of one animal of group A-controls that died 3 days after bacterial challenge and; (d) showing moderate activation of these areas of one animal of group B-administered clarithromycin that died 3.5 days after bacterial challenge.

Bacterial loads and pathology scores of the infected kidney, liver, spleen and lower lobe of the right lung when death occurred are shown in Table 3 and in Figure 3. Negative correlations were found between bacterial load and pathology scores of spleen of group B (r: -0.884, p: 0.047). Mean log_10_of viable bacteria of liver, spleen, kidney and lower lobe of the right lung of the two animals of group B that eventually survived were 1, 1, 2.73 and 1 respectively. Respective values for the three animals of group D that eventually survived were 1.62, 1, 2.78 and 1.97. Mean pathology scores of liver, spleen, kidney and lower lobe of the right lung of the two animals of group B that eventually survived were 0.5, 0, 1.5 and 0 respectively. Respective values for the three animals of group D that eventually survived were 0, 0, 0.33 and 0.

**Table 3:** Comparative bacterial load and pathology scores of tissues between groups of treatment of animals that died after bacterial challenge.

	**A (controls)**	**B (clarithromycin)**	**C (amikacin)**	**D (clarithromycin + amikacin)**
	
	**Bacterial load (mean ± SE, log_10 _of cfu/mL)**			
Liver	3.46 ± 0.79	3.34 ± 0.89	4.19 ± 0.92	2.14 ± 0.59
Spleen	3.64 ± 0.75	3.63 ± 0.92	4.10 ± 0.90	3.58 ± 0.79
Kidney	5.94 ± 0.50	4.92 ± 0.73	6.63 ± 0.30	6.82 ± 0.12
Lung	4.73 ± 0.59	4.09 ± 0.83	6.28 ± 0.39	5.76 ± 0.62
	**Pathology score (mean ± SE)**			
Liver	1.25 ± 0.25	0.38 ± 0.18^a^	0.80 ± 0.20	0.71 ± 0.28
Spleen	2.50 ± 0.38	2.70 ± 0.37	2.80 ± 0.20	2.43 ± 0.37
Kidney	2.38 ± 0.46	2.13 ± 0.52	2.30 ± 0.58	2.71 ± 0.36
Lung	1.38 ± 0.32	0.75 ± 0.25^b^	1.30 ± 0.16	1.29 ± 0.29

*P *of comparisons to group A: ^a^0.012, ^b^0.049

Mean ± SE concentrations of clarithromycin of group B at 3.5 και 24 hours were 5.44 ± 1.63 and 1.70 ± 0.59 μg/ml respectively. Respective concentrations of group D were 4.77 ± 0.86 and 2.17 ± 0.86 μg/ml (pNS compared to group B). Mean ± SE concentrations of amikacin of group C at 3.5 and 24 hours were 6.12 ± 1.69 and 0.39 ± 0.19 μg/ml respectively. Respective concentrations of group D were 7.68 ± 3.73 and 2.40 ± 1.50 μg/ml (pNS compared to group B).

## Discussion

Although immunomodulatory intervention is a very attractive approach for the management of severe sepsis, clinical attempts have failed. It is very probable that the design of animal studies before the initiation of these immunomodulators in clinical practice might be inadequate since in all animal models therapy is administered before induction of sepsis [1]. The present study focused on the application of clarithromycin as an immunomodulator upon presentation of signs of urosepsis by multidrug-resistant *P. aeruginosa*. Clarithromycin was administered intravenously in order to achieve serum levels close to 5 or 10 μg/ml; these concentrations are proposed as adequate for the inhibition of biosynthesis of pro-inflammatory cytokines by monocytes [4]. Amikacin was co-administered to simulate the clinical state of nosocomial sepsis often caused by multidrug-resistant species and where antimicrobials probably inactive on the offending pathogens are prescribed.

The advent of urosepsis was profound at the selected time interval of therapy, as evidenced by hypothermia, decrease of oxygen saturation and leucopenia (Figure 1). Administration of clarithromycin was accompanied by an increase of both rectal temperature and oxygen saturation and by prolonged survival (Figures 1 and 2). Amikacin did not have any effect neither on survival nor on the physical signs of animals. Co-administration of clarithromycin and amikacin allowed, however, for prolongation of survival to reach statistical significance.

The mode of action of clarithromycin might involve either a direct effect on the test isolate or its intervention on the inflammatory cascade. It has already been reported in animal models of chronic infection of the respiratory tract by *P. aeruginosa*, that clarithomycin administered before induction of the infection inhibited bacterial growth [12]. In vitro synergy with antipseudomonal β-lactams has also been described [13]. However, in the present study a direct effect of clarithromycin on the test isolate was unlikely since no effect was noted either on the number of bacteria in blood or in tissues (Table 3).

All groups of treatment were presented with the same extent of bacteremia and endotoxemia (Table 1) and thus with the same chance of triggering the inflammatory cascade. Administration of clarithromycin effectuated decrease of total oxidant status. That effect was attested by serum levels of MDA in animals of group B as well as by the area under the MDA curve in animals of group D. These findings are consistent with in vitro reports on the inhibition of neutrophil oxidative burst by clarithromycin [14].

In order to discriminate the mode of action of clarithromycin, blood monocytes were isolated from all animal groups and assayed both for rate of apoptosis and ex vivo release of TNFα (Table 2). Administration of clarithromycin either single or in combination with amikacin effected decrease of TNFα release by monocytes. In animals treated by single amikacin, a pro-apoptotic effect was noted at 48 hours. On the contrary, therapy by both clarithromycin and amikacin induced decrease of the total caspase-3 activity of monocytes. All these observations are consistent with a beneficiary effect of clarithromycin on the biology of blood monocytes.

The correlation between the presence of bacteria in organs and the respective pathology scores might be applied as an indirect criterion for the anti-inflammatory mode of action of clarithromycin. It is evident from Table 3 that bacteria in spleen and lung were not affected after the single administration of clarithromycin though their pathology score was decreased. Spleen is a major lymphoid organ where the presence of bacteria always stimulates an intense inflammatory reaction. The absence of such a reaction despite the presence of the offending pathogen, which is further indicated by the negative correlation between bacterial load and pathology scoring, is consistent with an anti-inflammatory mode of action of clarithromycin. (Figure 3 and Table 3).

Single clarithromycin exerted a per se immunomodulatory mode of action as evidenced by its beneficiary effect on vital signs and on the pathology scores of liver and spleen. Co-administration of clarithromycin and amikacin prolonged survival of septic animals and decreased blood oxidant status and monocyte apoptosis. The latter findings might be consistent with a synergistic immunomodulatory effect of both drugs.

## Conclusion

The present study clearly showed that clarithromycin administered intravenously upon advent of clinical signs of experimental sepsis by multidrug-resistant *P. aeruginosa *prolonged survival and ameliorated clinical findings. Its effect was most probably attributed to immunomodulatory intervention on blood monocytes. The successful results of this experimental study might be attributed to the altered pharmacokinetic regimen of clarithromycin allowing for serum levels higher than those achieved by the conventional oral doses. Once daily slow release oral formulations of clarithromycin deliver drug concentrations in the epithelial lining fluid of lung similar to those desired in the present study [15]. Further research should also target to intravenous administration of clarithromycin in order to elucidate the clinical significance of the reported experimental results for patients with sepsis.

## Abbreviations

FMOC: 9-fluorenylmethyloxycarbonyl chloride

LPS: endotoxins

MDA: malondiadehyde

TNFα: tumour necrosis factor-alpha

## Competing interests

The author(s) declare that they have no competing interests.

## Authors' contributions

EJGB participated in the design of the study, in the performance of estimation of inflammatory parameters and drafted the manuscript.

AA participated in the isolation of monocytes, the estimation of TNFα, LPS and caspase-3 and in tissue cultures

MR participated in the administration of the drugs and in the conduct of the animals' experiments

PK participated in the administration of the drugs and in the conduct of the animals' experiments

TT participated in the estimation of MDA and drug levels

VV performed tissue histology

CP participated in tissue and blood cultures

TA participated in the conduct of the animals' experiments

HG participated in study design and drafted the manuscript

## Pre-publication history

The pre-publication history for this paper can be accessed here:



## References

[B1] Vincent JL, Sun Q, Dubois MJ (2003). Clinical trials of immunomodulatory therapies in severe sepsis and septic shock. Clin Infect Dis.

[B2] Arndt P, Abraham E (2001). Immunological therapies for sepsis. Intensive Care Med.

[B3] Giamarellos-Bourboulis EJ, Adamis T, Laoutaris G, Sabracos L, Koussoulas V, Mouktaroudi M, Perrea D, Karayanncos PE, Giamarellou H (2004). Immunomodulatory clarithromycin treatment of experimental sepsis and acute pyelonephritis caused by multidrug-resistant *Pseudomonas aeruginosa*. Antimicrob Agents Chemother.

[B4] Kikuchi T, Hagiwara K, Honda Y, Gomi K, Kobayashi T, Takahashi H, Tokue Y, Watanabe A, Nukiwa T (2002). Clarithromycin suppresses lipopolysaccharide-induced interleukin-8 production by human monocytes through AP-1 and NF-kappa B transcription factors. J Antimicrob Chemother.

[B5] National Committee for Clinical and Laboratory Standards (2004). Performance standards for antimicrobial susceptibility testing 14th International supplement 24.

[B6] Frendéus B, Godaly G, Hang L, Karpman D, Lundstedt AC, Svanborg C (2000). Interleukin 8 receptor deficiency confers susceptibility to acute experimental pyelonephritis and may have a human counterpart. J Exp Med.

[B7] Levy M, Fink MP, Marshall JC, Abraham E, Angus D, Cook D, Cohen J, Opal SM, Vincent JL, Ramsay G (2003). 2001 SCCM/ESICM/ACCP/ ATS/SIS international sepsis definitions conference. Crit Care Med.

[B8] Robaux MA, Dube L, Caillon J, Bugnon D, Kergueris MF, Navas D, LeConte P, Barron D, Potel G (2001). In vivo efficacy of continuous infusion versus intermittent dosing of ceftazidime alone or in combination with amikacin relative to human kinetic profiles in a *Pseudomonas aeruginosa *rabbit endocarditis model. J Antimicrob Chemother.

[B9] Engelhard D, Pomernz S, Gallily G, Strauss N, Tuomanen E (1997). Serotype-related differences in inflammatory response to *Streptococcus pneumoniae *in experimental meningitis. J Infect Dis.

[B10] Agarwal R, Chase SD (2002). Rapid, fluorometric-liquid chromatographic determination of malondialdehyde in biological samples. J Chromatogr.

[B11] Sastre Toraño J, Guchelar HJ (1998). Quantitative determination of the macrolide antibiotics eryrthromycin, roxithromycin, azithromycin and clarithromycin in human serum by high-performance liquid chromatography using pre-column derivatization with 9-fluorenylmethyloxycarbonyl chloride and fluorescence detection. J Chromatogr B.

[B12] Yanagihara K, Tomono K, Imamura Y, Kaneko Y, Kuroki M, Sawai T, Miyasaki Y, Hirakata Y, Mukae H, Kadota J, Kohno S (2002). Effect of clarithromycin on chronic respiratory infection caused by *Pseudomonas aeruginosa *with biofilm formation in an experimental murine model. J Antimicrob Chemother.

[B13] Bui KQ, Banevicius MA, Nightingale CH, Quintiliani R, Nicolau DP (2002). In vitro and in vivo influence of adjunct clarithromycin on the treatment of mucoid *Pseudomonas aeruginosa*. J Antimicrob Chemother.

[B14] Culic O, Erakovic V, Parnham MJ (2001). Anti-inflammatory effects of macrolide antibiotics. Eur J Pharmacol.

[B15] Tagaya E, Tamaoki J, Kondo M, Nagai A (2002). Effect of a short course of clarithromycin therapy on sputum production in patients with chronic airway hypersecretion. Chest.

